# Some Notes on Counterfactuals in Quantum Mechanics

**DOI:** 10.3390/e22030266

**Published:** 2020-02-26

**Authors:** Avshalom C. Elitzur, Eliahu Cohen

**Affiliations:** 1 Institute for Quantum Studies, Chapman University, Orange, CA 92866, USA; avshalom@iyar.org.il; 2 Iyar, The Israeli Institute for Advanced Research, POB 651, Zichron Ya’akov 3095303, Israel; 3 Faculty of Engineering and the Institute of Nanotechnology and Advanced Materials, Bar Ilan University, Ramat Gan 5290002, Israel

**Keywords:** quantum mechanics, counterfactuals, weak values, time-symmetry, retrocausality

## Abstract

Counterfactuals, i.e., events that could have occurred but eventually did not, play a unique role in quantum mechanics in that they exert causal effects despite their non-occurrence. They are therefore vital for a better understanding of quantum mechanics (QM) and possibly the universe as a whole. In earlier works, we have studied counterfactuals both conceptually and experimentally. A fruitful framework termed quantum oblivion has emerged, referring to situations where one particle seems to "forget" its interaction with other particles despite the latter being visibly affected. This framework proved to have significant explanatory power, which we now extend to tackle additional riddles. The time-symmetric causality employed by the Two State-Vector Formalism (TSVF) reveals a subtle realm ruled by “weak values,” already demonstrated by numerous experiments. They offer a realistic, simple and intuitively appealing explanation to the unique role of quantum non-events, as well as to the foundations of QM. In this spirit, we performed a weak value analysis of quantum oblivion and suggest some new avenues for further research.

## 1. Introduction: The Odd Causal Efficacy on Quantum Counterfactuals

When Interaction-Free Measurement (IFM) was first introduced [1], it employed a somewhat overdramatized illustration: a supersensitive bomb, which a single photon suffices to detonate, even by merely being reflected from its sensor. Suppose now that you are not sure whether such a bomb is still good (i.e., bad for its victim), capable of exploding. Because it is an expensive bomb, you want to test but not waste it. How, given that explosion is triggered even by the weakest interaction possible, can you make sure only that it can explode?

Quantum mechanics makes it possible. Place the bomb on one of the two paths of a Mach Zehnder interferometer (MZI), e.g., attach it to one of the mirrors which is enabled to move, and send a single photon through. If the bomb is explosive, 25% of the cases (later raised towards 100% [2]) will result in the photon emerging from the MZI through the wrong exit, indicating that the bomb has measured the particle’s path. However, only an explosive bomb can function as a detector. Its detection is made not by a tiny click but by a violent explosion, yet the bomb will remain intact. Worse, if you know that the bomb is explosive, then once it does not explode, you are assured that the photon can be found only on the opposite MZI path. The riddle thus culminates: how can the photon on that path reveal not only the bomb’s presence on a distant path, but even indicate whether it functions?

Among the interpretations of QM, this challenge has been met with two opposite philosophies. The Many-Worlds interpretation (adhered by one of the IFM authors), as well as Bohmian mechanics, employ excessive ontology, invoking inaccessible parallel universes or equally unmeasurable nonlocal hidden variables, respectively, in order to account for the photon’s feat. In contrast, interpretations adhering to the Copenhagen school resorted to no ontology, namely the dismissal of any physical mechanism, arguing that quantum mechanics should deal with measurement data alone. 

We now propose a third option. The Two State-Vector Formalism (TSVF) [3,4,5] and related time-symmetric approaches [6,7,8,9,10,11,12,13,14] offer fresh insight into all the oddities of quantum mechanics, on the basis of the well-established time-symmetry of the quantum realm. Forward—and backward—evolving wavefunctions are granted equal causal efficacy. Consequently, effects proceed not only forward in time but may also zigzag back and forth. Hence, what appears to be nonlocal in space becomes local in spacetime. This account is therefore fully physical, i.e., compatible with all predictions of quantum mechanics and all experiments performed until now, independent of philosophical preferences.

Several diverse works of our group and colleagues based on this formalism, published during the last decade and fruiting into collaborations with some experimental groups, are beginning to converge into a novel and rigorous framework. While in full agreement with the standard quantum theory, this framework keeps revealing surprising layers of quantum reality. Moreover, it opens new possibilities for field theories [15] and cosmology [16,17,18]. In what follows, we present a brief and admittedly partial description of our recent advances along these lines, and conclude with some future goals.

## 2. The Key Phenomenon: Quantum Oblivion as the Basic Non-Event

**Figure 1:**
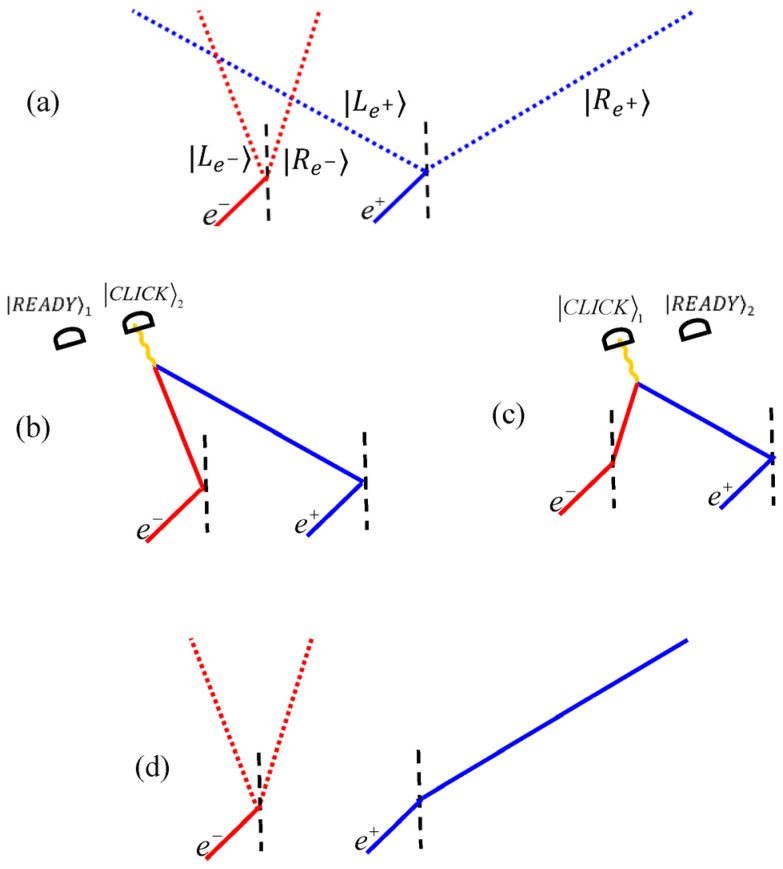
Possible electron–positron interactions and their outcomes. (**a**) The setting. (**b**,**c**) Annihilation. (**d**) Oblivion.

We begin with a very basic interaction [19] that offers a key for understanding quantum non-events. We let an electron and a positron pass through two Stern–Gerlach magnets (drawn as beam-splitters, Figure 1). The magnets split the particles’ paths according to their spins in the ***x***-direction:

(1) $$|\psi_{e^{-}}\rangle =\frac{1}{\sqrt{2}}\left(|L_{e^{-}}\rangle +|R_{e^{-}}\rangle \right)\;\mathrm{and}\;|\psi_{e^{+}}\rangle =\frac{1}{\sqrt{2}}\left(|L_{e^{+}}\rangle +|R_{e^{+}}\rangle \right)$$

Technical care is taken to ensure that, should the particles take intersecting paths, they would mutually annihilate. Two nearby detectors ${|READY\rangle }_{1}$ and ${|READY\rangle }_{2}$, are set to measure photons emitted upon pair annihilation, which would change their states to ${|CLICK\rangle }_{1}$ or ${|CLICK\rangle }_{2}$. 

Initially, the total wave-function is the separable state

(2) $$\left|\psi \right\rangle =\frac{1}{2}\left(|L_{e^{-}}\rangle +|R_{e^{-}}\rangle \right)\left(|L_{e^{+}}\rangle +|R_{e^{+}}\rangle \right){|READY\rangle }_{1}{|READY\rangle }_{2}$$

The particles, depending on their positions at times *t*_1_ or *t*_2_, may (not) annihilate and consequently (not) release a pair of photons, which would in turn (not) trigger one of the detectors. 

At $t_{0}\leq t<t_{1}$, then, the superposition is still unchanged as in Equation (2). However, at $t_{1}<t<t_{2}$, either a photon pair is emitted, indicating that the system ended up in $|R_{e^{-}}\rangle |L_{e^{+}}\rangle {|CLICK\rangle }_{1}{|READY\rangle }_{2}$, or not, and then:

(3) $$\left|\psi \right\rangle =\frac{1}{\sqrt{3}}\left[\left(|L_{e^{-}}\rangle +|R_{e^{-}}\rangle \right)|R_{e^{+}}\rangle +|L_{e^{-}}\rangle |L_{e^{+}}\rangle \right]{|READY\rangle }_{1}{|READY\rangle }_{2}$$

Similarly at $t>t_{2}$: If a photon pair is emitted, we know that the particles ended up in their left paths: $|L_{e^{-}}\rangle |L_{e^{+}}\rangle {|READY\rangle }_{1}{|CLICK\rangle }_{2}$. Otherwise, we find the non-entangled state:

(4) $$\left|\psi \right\rangle =\frac{1}{\sqrt{2}}\left(|L_{e^{-}}\rangle +|R_{e^{-}}\rangle \right)|R_{e^{+}}\rangle {|READY\rangle }_{1}{|READY\rangle }_{2}$$

which is an interesting result. The positron is observably affected: If we time-reverse its splitting, it may fail to return to its source. Its momentum has thus changed. Not so with the electron: It remains superposed, its reversibility being intact. 

This is quantum oblivion. Only one party “remembers” the interaction through momentum change, while the other remains oblivious. The apparent violation of Newton’s third law is resolved by the uncertainty principle, yet the resolution is highly nontrivial, leading to several new questions and applications [19,20,21,22]. 

Having pointed out this subtle type of interaction, we were able to show its heuristic power as underlying many quantum oddities considered unrelated so far [19,20,21,22]. These include, e.g., IFM, the Aharonov–Bohm effect (in the sense of temporary entanglement between the electron and solenoid [23,24,25]), the quantum Zeno effect (based on a dense sequence of counterfactuals followed by “forgetfulness” and re-initialization of the state), and, obviously, quantum erasure. In all these phenomena, a counterfactual event leaves visible causal traces despite its non-occurrence. A closer analysis, in the light of quantum oblivion, reveals more intriguing dynamics. The counterfactual event has actually occurred for a short time, and then “unoccurred” before measurement has finalized it into an actual non-event. Similarly, an entanglement has briefly formed between the interacting particles and then disappeared, leaving quantum correlations nevertheless. Consider again IFM: The photon and the bomb’s pointer have formed a momentary entanglement which was immediately dissolved, giving the impression that nothing has happened. Indeed, a subtler type of measurement, measuring the pointer’s position rather than momentum, can reveal this momentary entanglement. The latter option is, in fact, akin to weak measurement [26,27,28,29,30], which lies along the continuum spanned by quantum oblivion from no measurement to an ordinary projective measurement.

Returning to quantum oblivion, our question becomes sharper. Not only does the electron remain unchanged after the interaction that changed the positron’s momentum, but there are also the two remote detectors which took part in this non-reciprocal interaction—just by remaining silent. If no photon has ever been emitted from the electron–positron pair towards these detectors, located arbitrarily far away, how can their mere non-clicking affect the positron back?

From this analysis follow three paths of inquiry. One goes deeper into the theory in search of the physical dynamics enabling events to “unhappen” while leaving visible traces. The second path explores the ramifications of oblivion on a variety of physical processes, even at cosmological scales, on which it is likely to have non-trivial bearing. The third path explores applications of the above dynamics for quantum technology. 

## 3. TSVF Reveals the Underlying Dynamics

What then makes a quantum non-event? This is where TSVF sheds new light. It is a time-symmetric formalism, joining the transactional interpretation [6,7] and the Wheeler–Feynman model [31]. It ascribes a certain degree of retrocausality to quantum measurement, thereby highlighting several aspects of the nature of time, such as time’s arrow and reversibility. To the familiar forward-evolving wavefunction, TSVF adds a complementary backward-evolving wavefunction [4]. Their combination reveals a much richer quantum reality, based on the notion of weak values [22,32], as follows. 

First, when a quantum evolution is doubly computed this way, the knowledge gained from the two state-vectors together is greater than that gained from just one. When, e.g., the pre- and post-selected states are determined through the measurements of noncommuting operators, their variables simultaneously hold for the entire time interval between the past and future boundary conditions [4]. This apparently-forbidden knowledge holds only for the inaccessible past; hence the uncertainty principle is bypassed with no straightforward violation. Moreover, it was experimentally demonstrated that sequential weak measurements of two incompatible observables can be performed, thereby collecting information about both variables even at the single-particle level [33].

Let us generalize. Given an arbitrary pair of pre- and post-selected states, |ψ⟩ and |φ⟩, respectively, we can obtain, for any instance of time, the weak value of any operator ***A*** as $A_{w}=\langle \varphi \left|A\right|\psi \rangle /\langle \varphi |\psi \rangle$ by evolving the pre-/post-selected states forward/backward in time, respectively, until the relevant moment. Note that the weak value can reside even outside the spectrum of the measured operator, and may even be complex. Such complex weak values are practically helpful [34]. The gain obtained through weak measurements can become even greater when the pre- and post-selection form a rare combination. In these cases, the physical variables prevailing during the intermediate time interval can be very large, giving rise to weak value amplification [35,36], or superweak values [37]. In any case, the weak value gives rise to an effective interaction with a pre- and post-selected system as long as this interaction is weak enough [38]. Therefore, rather than being mere mathematical curiosities, TSVF shows that weak values have real ontological content. 

The common method to reveal these values used to be weak measurement. This technique changes the measured state only negligibly. One can therefore weakly measure several different operators, obtaining all the corresponding weak values as well as their temporal correlations which are related to Feynman’s sum over histories [39].

Recently, however, it turned out that even the standard “strong” quantum measurements can reveal these odd values. This can be done by indirect inference [40,41], and in some cases also by probe particles interacting with the system using quantum routers [42,43]. Hereinafter, we will focus on the latter approach. It should, however, be stressed that both methods of validation are immune to the statistical criticism against weak measurements [44,45,46].

The employed technique originates from a work by Aharonov and Vaidman [47], where it was shown how a single pre- and post-selected particle can simultaneously close two slits, such that another superposed particle is scattered off both slits, thereby maintaining its original superposition upon reflection. In terms of weak reality, the shutter particle separates right after the pre-selection into three quasi-classical particles, two occupying the two slits and one going into a third slit (through which we do not send the probe particle). As can be deduced from the weak values of the projection operators into these three places, two of these quasi-classical particles are “positive” (and therefore the probe particle is reflected from both) while the third quasi-classical particle is “negative”, a notion we previously described as a “counter-particle”. The total number of shutter particles thus remains one, but the pre- and post-selection allow a sharper resolution when we strongly detect the interference pattern of the reflected probe particle, and we find it to be intact. It is noted here that quantum superposition is revealed not only passively, in that both slits must be open for interference to occur, but also as an active state in which the superposed particle deflects the probe particle simultaneously off both its locations. If “strong claims require strong evidence,” this challenge is now fully addressed. 

**Figure 2:**
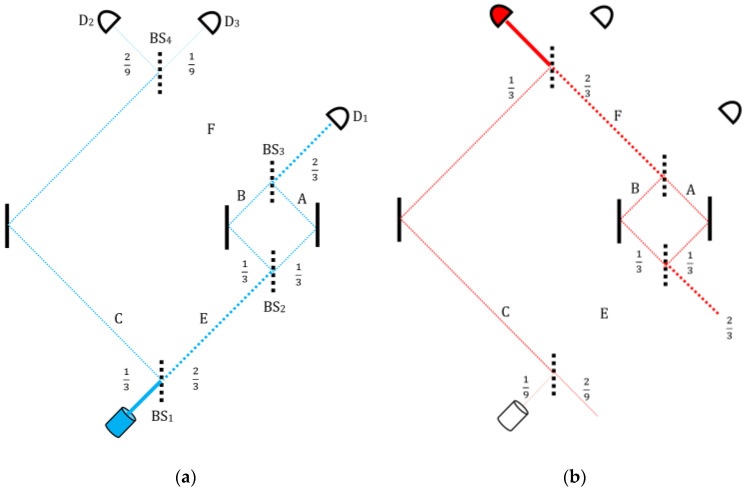

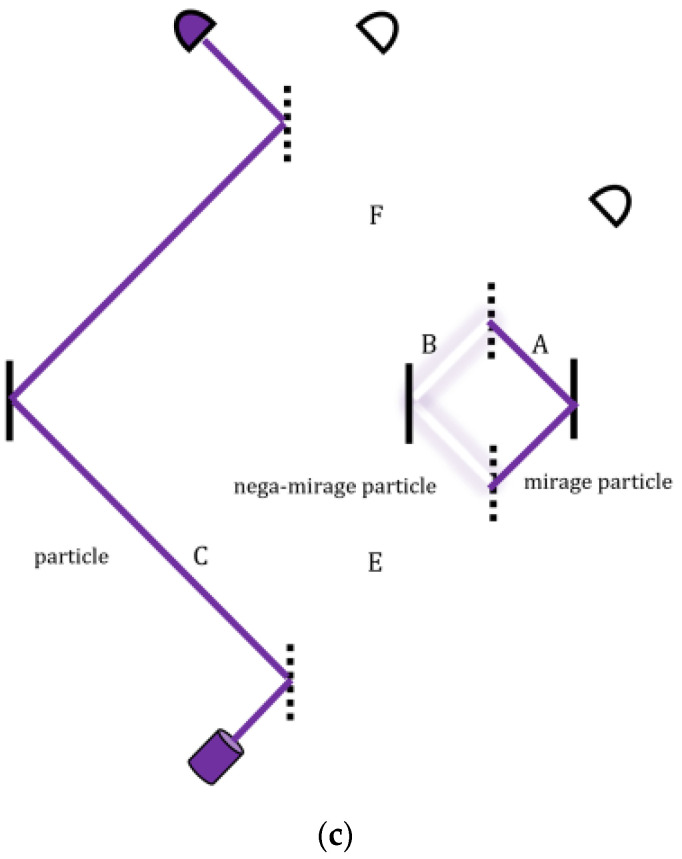
The two vectors taking place in the nested MZI and their joint prediction. (**a**) Forward state-vector (blue lines) with full and “empty” paths. (**b**) Backwards state-vector (red), again with full and “empty” paths. (**c**) The overlap between the pre- and post-selected states gives rise to an odd trajectory (purple) which harbors a short-lived particle in the middle of the “empty” path.

Such is also the case with the “Nested MZI”, originally due to Vaidman [48,49], and further elaborated by Aharonov et al. [22] for validation with projective measurement. A particle goes through an interferometer, within which a smaller one is nested (Figure 2). Upon the desired clicking and non-clicking of the appropriate detectors, it turns out that only one path of the large MZI has been traversed (solid red line), rendering the other, with the smaller MZI, empty (broken red). Similarly, for the particle’s backward-evolving history, there is one real past path (solid blue line) going backward and the other (broken blue) is empty. Lo and behold, within the small MZI, the overlap of the two empty histories gives rise to a segment which has been traversed (purple line) by a real short-lived particle.

With the gedanken aid of an auxiliary probe particle, Aharonov et al. [22] have shown that although no particle has entered the small MZI and neither has emerged from it, the short-lived particle has actually traversed it nonetheless, deflecting the probe particle sent to detect its presence. Laboratory confirmations from Kyoto University of this and related predictions are expected to appear soon. 

Even more intriguing is the mechanism by which this prediction has been derived. As shown straightforwardly in [22], the combination of the two state-vectors gives a negative value for the counter-particle along the middle-right segment of its past path, within the smaller MZI (Figure 2). If we choose to trust the mathematics and assign this minus sign to the counter-particle, then this sign turns out to effectively describe the particle’s potential interactions with other test particles, i.e., it pertains to the particle’s very presence.

This account offers a very simple and intuitive understanding of what has actually happened: The “empty” path of the large MZI, where apparently no particle has passed, was traversed by a pair of mirage particles, one with positive and the other with a negative mass. The smaller MZI briefly split them, before they reunited to form the apparent “nothing” again [50].

Supplementing the “strong” measurements with the familiar “weak” ones [22], we made the smaller MZI’s left mirror unfixed, such that it can indicate the particle’s passage by recoil. In accordance with the TSVF analysis, the momentum transferred to this mirror by the particle was negative; the mirror was pulled rather than pushed. Several other TSVF predictions involving such minus particles have been similarly verified with the aid of both weak and projective measurements. 

The answer to the question posed in the introduction is now at hand: a counterfactual event is not merely an event which did not occur but rather an occurrence accompanied by an unoccurrence through the self-cancellation of particles and counter-particles. 

## 4. Analysis of Quantum Oblivion with Weak Values

**Figure 3:**
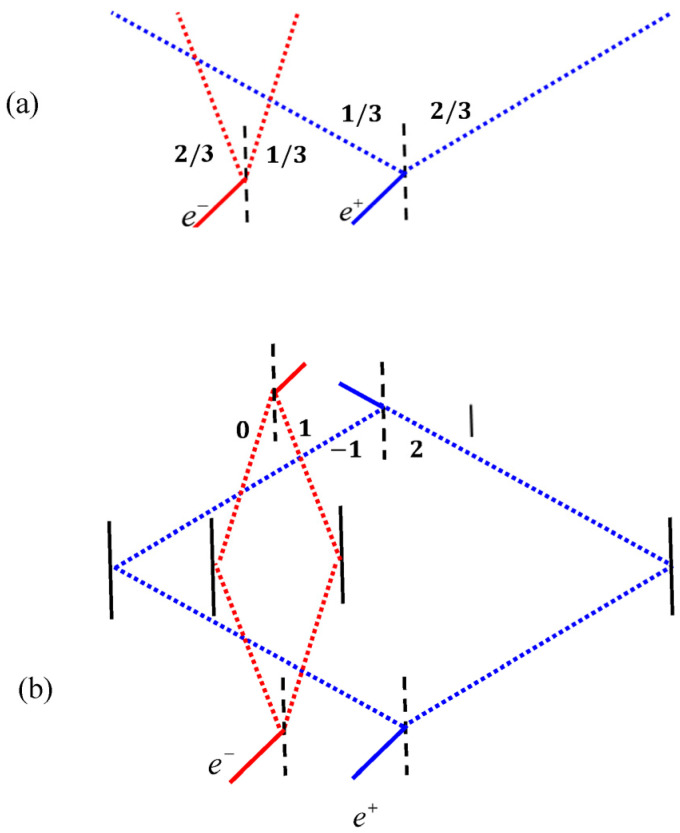
The oblivion experiment analyzed by the Two State-Vector Formalism (TSVF). (**a**) Weak values corresponding to the presence of the electron and positron at several positions are denoted in boldface for $t_{0}<t<t_{1}$ (**b**) Similarly, for the times before post-selection, in both cases anomalous weak values emerge which correspond to the whereabouts of the particles.

As is usually the case with quantum paradoxes, weak values enable a deeper understanding of quantum oblivion. Returning to the scenario in Figure 3a, let us take, as intermediate post-selection, the case of no-click at time *t*_1_. Then, at times $\;t_{0}<t<t_{1}$, the weak values of the projection operators are:

(5) $${\left(|L_{e^{-}}\rangle L_{e^{-}}|\right)}_{w}=\frac{2}{3},{\left(|R_{e^{-}}\rangle R_{e^{-}}|\right)}_{w}=\frac{1}{3},{\left(|L_{e^{+}}\rangle \langle L_{e^{+}}|\right)}_{w}=\frac{1}{3},{\left(|R_{e^{+}}\rangle \langle R_{e^{+}}|\right)}_{w}=\frac{2}{3}$$

The total number of particles is still 2, but we see a non-trivial splitting, reflecting the effective presence in each route in light of the post-selection.

If we now take, as the final post-selection, the state $\frac{1}{2}\left(|L_{e^{-}}\rangle +|R_{e^{-}}\rangle \right)\left(|L_{e^{+}}\rangle -|R_{e^{+}}\rangle \right)$, reflecting a perturbed interference (“collapse”) of the positron due to the electron’s presence, then we find an intriguing weak reality at the preceding times (see Figure 3b):

(6) $${\left(|L_{e^{-}}\rangle \langle L_{e^{-}}|\right)}_{w}=0,{\left(|R_{e^{-}}\rangle \langle R_{e^{-}}|\right)}_{w}=1,{\left(|L_{e^{+}}\rangle \langle L_{e^{+}}|\right)}_{w}=-1,{\left(|R_{e^{+}}\rangle \langle R_{e^{+}}|\right)}_{w}=2$$

This is counterintuitive; we thought that the electron was left unchanged, but the inability of the positron to maintain its initial superposition state (weakly) localizes the electron at the intersecting path. What is also non-trivial is the interplay of two quasi-classical particles and one counter-particle for describing the electron’s weak reality. This is in accordance with the electron’s tendency to reside in the right path, which moreover attaches to it a superweak value of 2, which has to be countered by a (−1) value in order to preserve the number of particles.

The correlations between the projectors complete the description:

(7) $${\left(|L_{e^{-}}\rangle \langle L_{e^{-}}||L_{e^{+}}\rangle \langle L_{e^{+}}|\right)}_{w}=-1,{\left(|L_{e^{-}}\rangle \langle L_{e^{-}}||R_{e^{+}}\rangle \langle R_{e^{+}}|\right)}_{w}=1$$

(8) $${\left(|R_{e^{-}}\rangle \langle R_{e^{-}}||L_{e^{+}}\rangle \langle L_{e^{+}}|\right)}_{w}=0,{\left(|R_{e^{-}}\rangle \langle R_{e^{-}}||R_{e^{+}}\rangle \langle R_{e^{+}}|\right)}_{w}=1$$

This allows us to understand the inexistence of the electron on the left path, ${\left(|L_{e^{-}}\rangle \langle L_{e^{-}}|\right)}_{w}=0$ via the aggregation of two self-cancelling weak values (Equation (7)). Note that the total number of pairs (sum of Equations (7) and (8)) is still 1, as it should be.

## 5. Recent Experiments Involving IFM

Counterfactuals are interesting not only to the theoretical physicist; they open up interesting technological applications as well. Last year, we had the privilege of collaborating with the Karimi group and additional colleagues in realizing a new imaging scheme we call interaction-free ghost imaging [51]. As the name implies, this scheme combines the benefits of quantum ghost imaging with those of interaction-free imaging in order to allow the imaging of structured objects (including birefringent ones) with reduced amounts of absorbed radiation, offering potential benefits when imaging light-sensitive substances. An “interaction-free interferometer”, containing the object to be imaged, is inserted into the path of one photon, while only its entangled companion eventually reaches a camera, correlated with the mere detection of the first photon. A reduction of 26.5% in the number of absorbed photons has been achieved while maintaining the same SNR of conventional ghost imaging. Conversely, an improvement of the SNR was shown, given the same amount of object illumination. This will hopefully pave the way for low-dose medical imaging, especially in the X-ray and gamma-ray regimes, a topic which we are now exploring. Interestingly, there is basic feasibility for demonstrating that, since recently ghost imaging with entangled X-ray photons was demonstrated [52]. What is also intriguing is the recent suggestion, supported by new technological developments, to perform interaction-free measurements with electrons [53,54,55].

The interaction-free ghost imaging scheme later led to another demonstration [56], this time of the nonlocal erasure of phase objects, which could be used for remote error correction and authentication. This, in fact, also demonstrates an imaging variant of partial measurements [57,58] which stemmed from IFM as well.

## 6. Discussion: A Counterfactual Universe

To conclude, we discuss another prototypical scenario encapsulating the strength of counterfactuals. Consider an isolated atom somewhere in empty space, and suppose it is excited. A very long time can elapse before it emits a photon and even longer before the photon is absorbed by a macroscopic object very far away. The eventless time interval, we now realize, is replete with counterfactual emissions and absorptions with numerous absorbers, creating complex entanglement and other subtle effects despite their non-occurrence [59,60]. In fact, we know this for the apparently dead vacuum, which at the quantum level turns out to host numerous pair creations and annihilations between particles with positive and negative masses and energies. To this, TSVF now adds the layer of “empty” particle trajectories, traversed by similarly elusive pairs of quasi-particles of which one is a counter-particle. Such pairs sometimes carry physical properties of the particle so far considered intrinsic to it, like a spin traversing a path other than that of the particle itself [61]. The study of this subtle reality, with its theoretical insights and technological uses, is very promising. 

Next, consider the basic and troublesome notion of collapse, either in the form of an actual absorption or a counterfactual one as in IFM. The TSVF’s account for this event is similarly straightforward. During the time interval between quantum preparation (e.g., emission) and measurement (absorption), namely pre- and post-selections, respectively, the particle’s state harbours a large number of quasi-classical particles and counter-particles. As exotic as this may seem, these particles are rigorously derived from the standard time-symmetric formalism between the past and future boundary conditions. Among these quasi-classical particles, positive and negative ones eventually eliminate one another so that the total number, upon measurement, is always eventually one. Such dynamics have been vividly demonstrated in the above experiment of the disappearing and repairing particle involving a particle superposed over three distant locations [50]. This unique pattern is the straightforward consequence of the above elimination between positive and negative emerging particles. An experiment following this proposal is currently underway. 

The bearing of this formulation on the nature of quantum counterfactuals—in fact on QM in general—is now clearer. The random occurrence and unoccurrence of quantum events following measurement provide an explanation that is both natural and novel, while in full agreement with quantum theory. The exchanges of positive and negative weak values along both time directions open new vistas for study, both experimental and theoretical, that can hardly be overestimated. 

A number of theoretical offshoots merit further study into the foundations of physics. Our first aim is a generalization of the recent TSVF derivations for all quantum mechanical interactions. The above counter-intuitive results appearing in specially tuned pre- and post-selected systems should be present in more mundane situations also, albeit in a less idealized way. We are now seeking to employ TSVF such that it gives a coherent picture for the full landscape. 

A parallel line of investigation concerns macroscopic scales [62]. As stressed above, counterfactual quantum interactions in the universe outnumber the actual ones by far, especially for long-distance interactions at the cosmological scale. This calls for further research into those processes where these interactions are prevalent.
